# Association Between Daily Steps Measured by Accelerometry and Diabetes in ELSA-Brasil Participants

**DOI:** 10.3390/ijerph23030346

**Published:** 2026-03-10

**Authors:** Matheus Hortélio, Maria da Conceição Chagas de Almeida, Sheila Maria Alvim de Matos, Cristiano Penas Seara Pitanga, Ciro Oliveira Queiroz, Francisco José Gondim Pitanga

**Affiliations:** 1 Postgraduate Program in Rehabilitation Sciences, Multidisciplinary Institute for Rehabilitation and Health, Federal University of Bahia (UFBA), Salvador 40110-170, BA, Brazil; 2 Gonçalo Moniz Institute, Oswaldo Cruz Foundation, Salvador 40296-710, BA, Brazil; 3 Institute of Collective Health, Federal University of Bahia (UFBA), Salvador 40110-040, BA, Brazil; 4 Department of Physical Education, Catholic University of Salvador (UCSAL), Salvador 41740-090, BA, Brazil; 5 Department of Physiology and Human Sciences, Southwest Bahia State University (UESB), Jequié 45205-490, BA, Brazil

**Keywords:** diabetes, daily steps, epidemiology, accelerometer, cohort study

## Abstract

**Highlights:**

**Public health relevance—How does this work relate to a public health issue?**

- This study addresses a relevant public health issue by demonstrating that daily physical activity, measured in steps per day, is associated with a lower prevalence of diabetes in adults. A cutoff point of 6880 steps/day with a protective effect was identified, offering a simple metric for the population. With approximately 12,636 federal employees, the study presents good statistical robustness and external validity for urban work contexts. The findings align with WHO recommendations to increase daily movement to prevent chronic diseases and highlight the use of low-cost technologies such as pedometers and smartphones.
- These findings are relevant because they indicate that a feasible number of daily steps already exerts a protective effect against diabetes, making the recommendation more accessible to the population. The cutoff point of 6880 steps/day allows scientific evidence to be transformed into simple and objective messages. In a scenario of high prevalence of sedentary lifestyles and diabetes, the results support low-cost preventive strategies. The large sample size reinforces the epidemiological consistency of the findings. Therefore, the study also supports public policies aligned with the recommendations of the World Health Organization.

**Public health significance—Why is this work of significance to public health?**

- This study is important for public health because it demonstrates that adopting a realistic level of daily physical activity is associated with protection against diabetes. Defining a target cutoff point of 6880 steps/day facilitates communication of the recommendations to the population. Due to the large sample size, the findings have high epidemiological relevance. The results support simple, low-cost, and widely applicable interventions. In this way, they contribute to prevention policies aligned with the guidelines of the World Health Organization.
- Furthermore, walking is an accessible activity for most people, regardless of age, socioeconomic status, or place of residence. The study also provides scientific support for the formulation of public policies aimed at promoting active lifestyles. By reducing the incidence of diabetes, actions based on these findings can contribute to a decrease in complications associated with the disease. Consequently, there is a potential reduction in the demand for health services and in costs for the public system. Thus, this study provides evidence that strengthens sustainable strategies for health promotion and disease prevention at the population level.

**Public health implications—What are the key implications or messages for practitioners, policy makers and/or researchers in public health?**

- The study presents important messages for different public health stakeholders. For healthcare professionals, it indicates that daily step targets are a practical strategy for preventing diabetes. For managers, the cutoff point of 6880 steps/day can guide population-based physical activity promotion programs with low cost and easy monitoring, aligned with WHO guidelines. For researchers, it reinforces the importance of objective measures of physical activity and the need for longitudinal and interventional studies to confirm causality and adjust cutoff points in different populations.
- Furthermore, evidence-based public policies, such as campaigns to encourage active mobility and the use of monitoring devices, can reach large population groups. These actions also promote individual autonomy in self-care. Therefore, the results of this study reinforce the need to integrate the promotion of physical activity into national strategies to combat chronic non-communicable diseases.

**Abstract:**

Diabetes mellitus (DM) is a chronic disease characterized by hyperglycemia and alterations in lipid and protein metabolism. Daily step count, measured using accelerometers integrated into wearable devices with artificial intelligence support, represents an important indicator of physical activity for the prevention and management of DM when performed regularly at a minimum daily volume. Objective: We aimed to investigate the association between daily step count and DM and to determine the daily step cutoff point necessary to provide a protective effect among participants in the Longitudinal Study of Adult Health (LSAH). Methods: We performed a cross-sectional study that analyzed data from Wave 3 (2016–2018), including 12,636 participants. DM was the dependent variable, assessed by laboratory tests, and daily step count was the independent variable, measured by accelerometry. Associations were assessed using logistic regression, with odds ratios (ORs) and 95% confidence intervals (CIs). Results: A significant association was observed between daily step count and DM (OR = 0.76; 95% CI: 0.70–0.83). A cutoff point of 6880 steps/day was identified, with an area under the ROC curve of 0.58 (95% CI: 0.57–0.59). Conclusion: Daily step count appears to be associated with a protective effect against DM.

## 1. Introduction

Physical activity can be defined as any bodily movement produced by skeletal muscles that increases energy expenditure relative to rest. During exercise, increased glucose uptake by muscles can be 7 to 20 times the baseline level, reflecting greater energy utilization and increased insulin sensitivity in peripheral tissues [1]. For individuals with diabetes mellitus, regular physical activity offers significant benefits for metabolic control, including improved glycemic control and increased insulin action [2].

Different exercise modalities, such as aerobic and interval training, promote adaptations in skeletal muscle that increase glucose uptake and insulin sensitivity, and are widely recommended as an integral part of diabetes management [3].

Although physical activity is considered essential for the effective treatment of type 2 diabetes mellitus (T2DM), a considerable portion of this population remains physically inactive [4]. A study by Ekelund et al. [5] showed that small, realistic increases of 5 min per day in moderate to vigorous physical activity can prevent up to 6% of all deaths in a high-risk approach and 10% of all deaths in a population-based approach. Reducing sedentary time by 30 min per day can prevent a smaller, but still significant, proportion of deaths in both risk scenarios.

According to the new WHO guidelines, all adults should engage in 150 to 300 min of moderate-intensity physical activity or 75 to 150 min of vigorous-intensity physical activity, or some equivalent combination of both, per week. Among children and adolescents, an average of 60 min of moderate-to-vigorous aerobic physical activity per day throughout the week provides health benefits [6].

Although physical training can promote adaptations in skeletal muscle that increase the proportion of fibers with greater oxidative capacity (such as type IIa fibers), physical inactivity and insulin resistance tend to promote a more glycolytic muscle profile, with a higher prevalence of type IIx fibers. This change in muscle fiber phenotype is associated not only with differences in physical performance, but also with alterations in glucose metabolism, since different fiber types vary in their capacity for glucose uptake and utilization [7]. Chronic energy imbalance and inactivity contribute to muscle adaptations that impair insulin response, including lower glucose uptake and reduced glycogen synthesis in skeletal muscle in individuals with T2DM, reflecting the insulin resistance characteristic of the disease [8].

Reduced mitochondrial function and, consequently, decreased lipid oxidation capacity are additional characteristics of insulin-resistant individuals. When combined with increased systemic lipid influx, this condition facilitates ectopic lipid accumulation in skeletal muscle. Regular exercise can improve mitochondrial function in muscle cells and enhance fatty acid oxidation capacity [9]. Importantly, even a single bout of exercise is a potent stimulus for increasing glucose disposal by activating 5′-AMP-activated protein kinase (AMPK), which leads to the translocation of the insulin-independent glucose transporter GLUT4 and enhances glucose uptake and glycogen storage, thereby rapidly attenuating the metabolic abnormalities associated with DM2 [10].

Furthermore, a lack of regular physical activity can contribute to abdominal obesity, a state of excess central fat accumulated in the trunk and abdomen, implying a greater amount of visceral adipose tissue, which is metabolically active and strongly associated with an increased risk of metabolic diseases such as type 2 diabetes, hypertension, and dyslipidemia. It is typically assessed through anthropometric measurements such as waist circumference (e.g., values > 88 cm in women and >102 cm in men according to some criteria) or waist-to-height ratios. Recent literature emphasizes that these measurements capture the fat distribution that is mechanistically linked to insulin resistance and systemic inflammation, central mechanisms in the pathophysiology of chronic non-communicable diseases [11].

Wearable sensors based on artificial intelligence (AI), such as accelerometers, can collect, analyze, and transmit real-time health data to healthcare professionals, allowing for more efficient decision-making based on patient information. In this context, wearable sensors have become increasingly popular due to their ability to provide a non-invasive and convenient means of monitoring health status. These devices can monitor various health parameters, including physical activity levels measured by daily step counting, with AI support through wearable technologies [12].

Studies of the general population indicate an average frequency of 7000 to 13,000 steps per day in apparently healthy adults with an average age of 52 years (±5.3 years). In the population with diabetes mellitus (DM), studies have indicated an average of 6291 steps per day [13]. This suggests that DM can lead to a reduction in habitual physical activity through daily walking. Therefore, it is important to assess and analyze the habitual physical activity level of people with DM. Regular physical activity is a strategy recommended by several studies and guidelines for the control of DM2 and its associated complications [14].

A prospective study conducted at the University of California, San Diego, San Diego, CA, on elderly women who used accelerometers to measure daily steps showed that taking more steps per day was associated with a lower risk of developing DM2 over time, and the association was stronger for moderate-to-vigorous-intensity steps than for light steps. Each increment of 2000 steps/day was related to a lower risk of diabetes. This evidence corroborates the importance of regular walking as a risk factor for the prevention of DM2 in older adults [15].

In the Department of Community Medicine and Rehabilitation, Geriatric Medicine Unit, Umeå University, Umeå, Sweden, a prospective study of 3055 elderly people, aged 70 years and older, without diabetes at the start of the study measured steps with accelerometers for one week. The results show an inverse dose–response relationship between the number of steps per day and the risk of developing DM, and suggest that people taking ≥4500 steps/day had a lower risk of diabetes compared to those who walked less [16].

It is estimated that approximately 382 million individuals worldwide have DM, with projections indicating an increase to 471 million by 2035 [17]. In Brazil, the prevalence of DM varies from 0.9% among individuals aged 18 to 29 years to 14.5% among those aged 60 to 64 years, reaching 19.9% in individuals aged 65 to 74 years [18]. In the ELSA-Brasil study, after a follow-up period of 8.2 years, from baseline (2008–2010) to the second follow-up (2017–2019), Birck et al. (2022) [19] reported a DM incidence of 14.3%.

Individuals with DM have a substantial impact on public health spending. The average quarterly cost is approximately R$ 1212.37 for individuals with diabetes and vascular complications and R$ 931.88 for those without complications, with significantly higher costs observed during hospitalizations [20]. In addition to economic costs, DM also represents a significant social problem. Individuals affected by this disease frequently experience reduced quality of life, life expectancy, and mobility, leading to psychological, physical, and metabolic impairments [21]. Consequently, studies addressing strategies for DM prevention are highly relevant.

Therefore, the objective of this study was to investigate whether there is an association between the number of daily steps and DM and to identify the ideal cutoff point for daily step counting as a discriminator of the absence of DM.

## 2. Materials and Methods

### 2.1. Population and Sample

This cross-sectional study analyzed participants from the second follow-up of the ELSA-Brasil cohort (Longitudinal Study of Adult Health). Initiated in 2008 by the Ministry of Health, ELSA-Brasil is the largest epidemiological study in Latin America, investigating chronic diseases such as diabetes mellitus and cardiovascular diseases and their risk factors in the Brazilian population. ELSA-Brasil is also the largest multicenter cohort study conducted by a developing country and comprises six teaching and research institutions in different Brazilian cities (Salvador, Porto Alegre, São Paulo, Rio de Janeiro, Belo Horizonte, and Vitória). This study included 15,105 public servants from the six aforementioned institutions, aged between 35 and 74 years, at baseline. Among other tasks, they used an accelerometer and a sleep diary to measure their levels of physical activity and daily steps. To date, data have been collected at three time points: baseline (Wave 1), conducted between 2008 and 2010, the 1st follow-up (Wave 2) between 2012 and 2014, and the 2nd follow-up (Wave 3) between 2016 and 2018 [22,23,24]. Data for the 3rd follow-up (Wave 4), conducted between 2022 and 2024, are currently being collected. For the present study, all participants from the 2nd follow-up, Wave 3 (2016–2018), who had data collected using accelerometry were selected.

The ELSA-Brasil study was approved by the National Research Ethics Committee (CONEP MS 976/2006) and by all Research Ethics Committees of the six research centers involved. All participants signed an informed consent form, guaranteeing the confidentiality and secrecy of the data.

### 2.2. Data Production

The data was collected by a team of interviewers and measurers trained and certified by a quality control committee [25], qualified to execute the study protocol at any ELSA-Brasil Research Center.

Strict protocols were implemented for the initiation and downloading of the accelerometers.

### 2.3. Diabetes Assessment

Diabetes-related data were obtained following an overnight fasting period. Biological samples were collected from all participants, immediately processed and stored under freezing conditions, and subsequently transported to the central laboratory at the University of São Paulo. To ensure analytical consistency, all biochemical measurements were conducted at a single research facility. Diabetes mellitus was defined based on the following criteria in accordance with the guidelines the World Health Organization (2016): fasting plasma glucose (FPG) ≥ 126 mg/dL (7.0 mmol/L), 2 h plasma glucose (2hPG) ≥ 200 mg/dL (11.1 mmol/L) during a standard oral glucose tolerance test (OGTT), or glycated hemoglobin (HbA1c) ≥ 6.5% (48 mmol/mol) [21]. We considered a case prevalent at baseline when any of these criteria were present. Laboratory parameters were obtained by blood sampling after a 12 h overnight fast [26].

### 2.4. Assessment of Daily Steps

For daily step assessment, participants used an ActiGraph wGT3X-BT device (version 3.2.1, Actigraph Corp, Pensacola, FL, USA). We activated the devices in the ActiLife software (version 6.13.4, ActiGraph) with a sampling frequency of 30 Hz, recording triaxial acceleration in raw format. Participants wore the accelerometer attached with an elastic band above the right anterior superior iliac spine for 24 h for 7 consecutive days, starting at 8 p.m. on the same day as the study clinical consultation (day 1) and ending at 8 p.m. on day 8 (day 8). They were instructed to wear the device at all times but to remove it during aquatic activities such as swimming or bathing. Each participant’s data were validated if all of the following criteria were met: (1) presence of recorded data at each epoch of the sample’s 24 h cycle, even when combining different days; (2) a calibration error of less than 0.02 g after the post-measurement self-calibration process; (3) at least 4 days with 16 h or more of use; and (4) at least one of the 4 days being Saturday or Sunday [27]. Furthermore, the algorithm did not count physical activity for seated physical exercises, such as weight training and indoor cycling, nor activities performed in an aquatic environment. It also did not measure physical activity based on heart rate. Through the ROC curve, the cutoff point for the number of daily steps was determined, where sensitivity and specificity showed a maximum equilibrium point at 6880 steps per day, for both men and women. The algorithm used was that of Troiano.

### 2.5. Data Analysis

Descriptive measures (proportions) were calculated for all categorized variables. All analyses were stratified by sex a priori. Associations between the dependent variable (diabetes) and the independent variable (number of daily steps) were analyzed using logistic regression. The following variables were considered as potential confounders or effect modifiers: age, sex, obesity, abdominal obesity, family income, education, and smoking. Effect modification analysis was performed by examining stratum-specific point measures and their confidence intervals. If the point measure of a factor in a specific stratum is not in the confidence interval of another factor in the same stratum, this indicates effect modification. Confounding analysis was performed by comparing the odds ratio (OR) for the crude association with that for the association adjusted for potential confounders. The parameter used to identify the difference between the associations was 10%. Next, a logistic regression analysis was performed. The analysis began with a full model, followed by a one-by-one removal of each potential confounding variable. Subsequently, the OR (odds ratio) was estimated for the association between the number of daily steps and diabetes, and then stratified and adjusted for the variables that were confirmed as effect modifiers and confounders, respectively. The covariates abdominal obesity and leisure-time physical activity were identified as effect modifiers. No covariates were identified as confounders. The confidence interval was set at 95%.

The cutoff points for the number of daily steps for the absence of diabetes were identified using Receiver Operating Characteristic (ROC) curves, which are frequently used to determine cutoff points in diagnostic or screening tests (Erdreich & Lee, 1981) [28]. The statistical program STATA 17.0 was used.

## 3. Results

A total of 12,636 participants were included in the analysis, of which 5623 (44.50%) were men and 7013 (55.50%) were women, where men over 60 years old represent 56.21% while women over 60 years old represent 43.79%. An association was found between daily steps and diabetes (OR = 0.76, CI = 0.70–0.83), in addition to the cutoff point of 6880 with area under the ROC curve = 0.58 (CI = 0.57–0.59). After the analysis of effect modification and confounding, it was found that, in men, there was an association with statistical significance for those with abdominal obesity (OR = 0.62, CI = 0.43–0.87) and who performed moderate/vigorous leisure-time physical activity (OR = 0.64, CI = 0.50–0.80). In women, an association was observed only in those with abdominal obesity (OR = 0.86, CI = 0.75–0.98). The sample characteristics are presented in Table 1. The DM rate in women was 23.3%, while in men it was 29.0%. Men presented higher rates of obesity (60.79%) and abdominal obesity (66.69%) compared to women, who, in turn, presented higher rates of smoking (51.52%), moderate/vigorous physical activity in leisure time (51.38%), higher income (54.56%), presence of diabetes (50.08%), and a higher number of daily steps (52.84%). Men predominate in both the Black/mixed-race (55.69%) and White (54.97%) populations compared to women; however, both sexes are predominantly White.

Table 2 shows the association between the number of daily steps and diabetes adjusted for age and stratified by sex, abdominal obesity and leisure-time physical activity. When analyzing this combination, it was found that there was epidemiological relevance only in men with abdominal obesity who had more than 6880 steps per day. In the other associations, there was no relevance.

Table 3 shows the association between the number of daily steps and diabetes adjusted for age and stratified by sex and abdominal obesity. This combination showed epidemiological relevance only in women with abdominal obesity who took more than 6880 daily steps. The other associations were not relevant.

Table 4 shows the association between the number of daily steps and diabetes adjusted for age and stratified by sex and leisure-time physical activity. A significant association was identified only in men who had more than 6880 steps per day with moderate/vigorous physical activity during leisure time.

## 4. Discussion

This study included 15,105 public servants from the six institutions mentioned, aged between 35 and 74 years, at baseline. Among other tasks, they used an accelerometer and a sleep diary to measure their levels of physical activity and daily steps. To date, data have been collected at three time points: baseline (Wave 1), conducted between 2008 and 2010; first follow-up (Wave 2), between 2012 and 2014; and second follow-up (Wave 3), between 2016 and 2018 (DECIT, 2009; Eickemberg et al., 2020; Pitanga et al., 2021) [22,23,24]. Data for the third follow-up (Wave 4), conducted between 2022 and 2024, are currently being collected. For the present study, all participants from the second follow-up, Wave 3 (2016–2018), who had data collected using accelerometry were selected.

The present study aimed to investigate whether there is an association between the number of daily steps and DM and to identify the optimal cutoff point for daily step count as a discriminator for the absence of DM. Abdominal obesity can be defined as excessive fat accumulation in the abdominal region and is a health condition positively associated with non-communicable diseases [29]. Moreover, it is considered a particularly adverse form of obesity with serious health implications and has been strongly associated with common chronic non-communicable diseases, including cardiovascular disease, DM, hypertension, cancer, kidney disease, and fatty liver disease [29].

Cavero-Redondo et al. [30] conducted a meta-analysis confirming the beneficial effects of physical activity interventions on glycemic control, measured by glycated hemoglobin, in non-diabetic populations. In addition, Zhao et al. [31] reported that higher levels of physical activity were associated with a lower prevalence of DM, with a more pronounced risk reduction observed at lower baseline activity levels. Physical activity reduces the risk of DM through both short- and long-term improvements in insulin action, resulting in better glucose control [32].

Similar findings were reported in a study conducted in Japan in 2023 [33], which examined associations between glycemic variability, assessed using continuous glucose monitoring, sleep quality, and daily step counts measured by wearable devices in healthy individuals. The results demonstrated that glycemic variability was lower on days with higher daily step counts compared with days with lower step counts in both sexes. Given that positive correlations have been reported between glycemic variability and markers of oxidative stress [34] as well as coronary plaque rupture [35], increased glycemic variability may contribute to the development of cardiovascular complications [36]. Furthermore, glycemic variability has also been associated with sleep quality and physical activity. Poor sleep quality has been linked to increased glycemic variability in individuals with type 1 diabetes and DM2 [37].

Consistent with the present findings, Kim et al. [38] conducted a 12-week randomized, open-label, controlled, single-center extension study involving individuals with DM2 who had HbA1c levels <8.5% and a body mass index ≥23 kg/m^2^. All participants used a smartphone-based personal health record application. Regarding glycemic outcomes, the intervention group showed significantly lower HbA1c levels at week 12 compared with the control group, as well as significant between-group differences in changes in fasting glucose levels from baseline.

A systematic review by Hall et al. [39] reported robust evidence from two large studies with five-year follow-up periods that examined the association between daily step counts and the incidence of dysglycemia or DM2. These findings are consistent with the present study, demonstrating a significantly reduced risk associated with higher daily step counts. The authors concluded that accumulating fewer than 10,000 steps per day is sufficient to confer meaningful health benefits.

Data collected between 2010 and 2020 remain relevant in 2026 because they offer an essential historical basis for identifying long-term trends, behavioral changes, and the evolution of measurement technologies that are not captured by more recent data alone. Research in health and technology, for example, shows that the pattern of technology use by older populations gradually changes, and understanding these past patterns is crucial for interpreting current data and planning effective interventions in digital health and healthy aging. Methodological studies emphasize that retrospective analyses help project future trends and validate predictive models, since patterns observed a decade ago can have effects that last for more than ten years and influence subsequent scientific and technological development. Furthermore, contemporary advances in big data analytics and statistical methods allow for the comparison and integration of long time series, highlighting how user behaviors and technology adoption evolve over time, which increases the robustness of current conclusions [40].

## 5. Conclusions

Regular exercise involving repeated muscle contractions therefore provides the physiological basis for metabolic health and for the prevention and treatment of DM2. In this context, a potential relationship exists between daily step count and diabetes prevention, as daily steps can be considered a proxy for regular physical activity. Through similar physiological mechanisms, daily step count may act as a protective factor against DM, particularly among individuals with abdominal obesity.

This study has some limitations. First, the accelerometer measured the total daily step count without differentiating between intensity or step speed, making it impossible to determine whether the steps were accumulated at a faster or slower pace, which may affect the interpretation of the results. Second, although the sample included 15,105 participants at baseline and 12,636 participants in the present analysis, from three major regions of Brazil (Northeast, Southeast, and South) and reflecting regional and social diversity, the ELSA-Brasil cohort may not be fully representative of the Brazilian population, since it is mainly composed of public servants with relatively high levels of education and income. Third, despite the small area under the ROC curve, the confidence intervals allow for estimating the cutoff point. Furthermore, as this is a cross-sectional study, it is not possible to identify a causal relationship between the number of daily steps and DM. There were also no confounding factors in the study.

Based on the results of this study, exceeding 6880 daily steps may be associated with a lower risk of developing DM. Among individuals with abdominal obesity, a higher daily step count appears to be particularly important as a protective factor against chronic diseases. Furthermore, because it was not possible to assess step intensity, moderate-to-vigorous leisure-time physical activity seems to play a key role in DM prevention. Thus, within the limitations of the study, moderate- and vigorous-intensity leisure-time physical activity appears to be more strongly associated with DM protection than daily step count alone in both sexes.

The findings of this study may assist public health managers by reinforcing the importance of physical activity as a tool for preventing chronic diseases such as DM2. In addition, by suggesting a daily step-count target, these results may help inform public policies aimed at promoting walking by recommending distances that correspond to a minimum number of steps per day. However, despite the robustness of the present findings, further research is needed to clarify the effects of daily step-based interventions on DM-related outcomes.

## Figures and Tables

**Table 1:** Sample characteristics at Wave 3 of the ELSA-Brasil study (2016–2018).

Variables	Men, n (%)	Women, n (%)	*p*-Value
Age (years)			
41–59	3923 (54.95)	3216 (45.05)	0.157
>60	3090 (56.21)	2407 (43.79)	
Obesity			
No	4823 (53.59)	4176 (46.41)	<0.001
Yes	2105 (60.79)	1358 (39.21)	
Smoking status			
Never smoked	4578 (60.06)	3045 (39.94)	<0.001
Former/current smoker	2403 (48.48)	2554 (51.52)	
Leisure-time physical activity			
Light	5043 (58.72)	3545 (41.28)	<0.001
Moderate-to-vigorous	1896 (48.62)	2004 (51.38)	
Income (BRL)			
<6558.5	1882 (42.74)	2521 (57.26)	0.004
≥6558.5	3741 (45.44)	4492 (54.56)	
Abdominal obesity			
No	2688 (44.09)	3408 (55.91)	<0.001
Yes	4084 (66.69)	2040 (33.31)	
Race/ethnicity			
Black/Brown	3047 (55.69)	2424 (44.31)	0.427
White	3600 (54.97)	2949 (45.03)	
Glycated hemoglobin			
<6.5%	6075 (56.55)	4667 (43.45)	<0.001
≥6.5%	938 (49.52)	956 (50.48)	
Diabetes prevalence			
No	3891 (42.53)	5258 (57.47)	<0.001
Yes	1592 (49.92)	1597 (50.08)	
Daily step count			
<6880 steps/day	1895 (40.05)	2836 (59.95)	<0.001
≥6880 steps/day	3728 (47.16)	4177 (52.84)	

Data are presented as number (percentage). *p*-values obtained using the chi-square test.

**Table 2:** Association between daily step count and diabetes, adjusted for age and stratified by sex, abdominal obesity, and leisure-time physical activity.

Daily Step Count	No Abdominal Obesity Insufficient LTPA OR (95% CI)	No Abdominal Obesity Moderate-to-Vigorous LTPA OR (95% CI)	Abdominal Obesity Insufficient LTPA OR (95% CI)	Abdominal Obesity Moderate-to-Vigorous LTPA OR (95% CI)
Men				
<6880 steps/day	1.00 (Reference)	1.00 (Reference)	1.00 (Reference)	1.00 (Reference)
≥6880 steps/day	1.02 (0.81–1.28)	0.86 (0.61–1.22)	1.04 (0.83–1.29)	0.62 (0.43–0.87)
Women				
<6880 steps/day	1.00 (Reference)	1.00 (Reference)	1.00 (Reference)	1.00 (Reference)
≥6880 steps/day	1.22 (0.90–1.65)	1.15 (0.69–1.92)	0.90 (0.77–1.06)	0.78 (0.57–1.06)

LTPA: Leisure-time physical activity. Insufficient LTPA refers to the lowest level of physical activity. Moderate-to-vigorous LTPA was defined according to IPAQ criteria (≥600 MET-min/week). OR: Odds ratio; CI: confidence interval. Models adjusted for age.

**Table 3:** Association between daily step count and DM, adjusted for age and stratified by sex and abdominal obesity.

Daily Step Count	OR	95% CI	OR	95% CI
Men				
<6880 steps/day	1.00	Reference	1.00	Reference
≥6880 steps/day	0.94	0.78–1.14	0.87	0.72–1.04
Women				
<6880 steps/day	1.00	Reference	1.00	Reference
≥6880 steps/day	1.18	0.92–1.53	0.86	0.75–0.98

OR: Odds ratio; CI: confidence interval. Models adjusted for age.

**Table 4:** Association between daily step count and DM, adjusted for age and stratified by sex and leisure-time physical activity.

Daily Step Count	OR	95% CI	OR	95% CI
Men				
<6880 steps/day	1.00	Reference	1.00	Reference
≥6880 steps/day	0.89	0.77–1.03	0.64	0.50–0.80
Women				
<6880 steps/day	1.00	Reference	1.00	Reference
≥6880 steps/day	0.91	0.80–1.04	0.78	0.61–1.00

OR: Odds ratio; CI: confidence interval. Models adjusted for age.

## Data Availability

The data generated by the studies are the property of ELSA-Brazil and are not publicly available.
